# The Rigid Adsorbent Lattice Fluid Model: Thermodynamic Consistency and Relationship to the Real Adsorbed Solution Theory

**DOI:** 10.3390/membranes12101009

**Published:** 2022-10-18

**Authors:** Stefano Brandani

**Affiliations:** School of Engineering, University of Edinburgh, The King’s Buildings, Mayfield Road, Edinburgh EH9 3FB, UK; s.brandani@ed.ac.uk

**Keywords:** multicomponent adsorption, thermodynamic consistency, adsorbed solution theory

## Abstract

The Rigid Adsorbent Lattice Fluid model has been shown to comply with all the requirements for thermodynamic consistency in the case of an adsorbent that does not undergo structural changes. This is achieved by introducing a correction to the reduced density function that multiplies the combinatorial term. A procedure to calculate the predicted adsorbed mixture activity coefficients has been presented that allows the production of excess Gibbs energy plots at a constant reduced grand potential. The predicted nonideality is structurally consistent with the Non-Ideal Adsorbed Solution Theory of Myers in terms of both its dependence on concentration and reduced grand potential. The ability to generate excess Gibbs energy values allows linking the new Rigid Adsorbent Lattice Fluid model to the traditional Real Adsorbed Solution Theory providing an alternative approach to predicting multicomponent adsorption based solely on pure component data.

## 1. Introduction

A new thermodynamic framework for pure and mixed gas adsorption, the Rigid Adsorbent Lattice Fluid (RALF) model was presented in [1], adapting to a non-distributing solid the Non-Equilibrium Lattice Fluid (NELF) approach for polymers developed and applied extensively by Professor Sarti and his colleagues at the University of Bologna [2,3,4,5], including a recent extensive review [6]. Since its introduction, which considered the adsorption of gases and vapours in the zeolite silicalite, the RALF model has been used to correlate adsorption in breathing metal–organic frameworks [7]; to demonstrate that it allows predicting different types of isotherms depending on the physical parameters of the adsorbates [8]; and more recently, it was shown to capture the behaviour of ZSM-25 in dynamic experiments [9] as well as water adsorption with stepped behaviour [10]. The emphasis of these publications [1,7,8,9,10] has been to investigate the applicability of RALF to a range of challenging adsorption systems as well as test the prediction capability for multicomponent adsorption, thus focusing primarily on the phase equilibria of the adsorbates.

The purpose of the present contribution was to study in greater detail the case of the frozen solid, where the adsorbent does not undergo structural changes, and focus on:

1. The requirements for thermodynamic consistency, which as will be shown lead to a small modification to the adsorbed phase expression for multicomponent mixtures.

2. Demonstrate how it is possible to determine the adsorbed phase excess Gibbs energy from RALF, thus providing the means to bridge this approach to the classical Real Adsorbed Solution Theory (RAST).

It is hoped that this contribution will be of interest to both adsorption and membrane communities that have developed thermodynamic approaches for phase equilibria between the gas (and liquid) and solid phases with limited attempts at reconciling the apparently different approaches. One would hope as well that this will not lead to a sense of bewilderment in the reader [6] as in both cases the assumption made is that the solid phase (membrane or adsorbent) is considered to be a lattice fluid.

## 2. RALF Model for a Frozen Adsorbent

A basic assumption in the thermodynamics of adsorption is that the adsorbent does not undergo structural changes [11] and in the RALF framework this corresponds to the frozen solid limit [1]. The volume of the system, V, is assumed to be the volume occupied by the solid, including the micropores, $V_{S}$, [12] and

(1) $$V=V_{S}=\frac{m_{S}}{\rho_{S}}=\frac{\sum_{j}m_{j}}{\rho }$$

where $\rho_{S}$ is the density of the solid including the micropores, ρ is the density of the adsorbed phase, including the adsorbates. $m_{S}$ is the mass of the solid, while $m_{j}$ are the masses of all components, including the solid. In the frozen solid limit $V_{S}$ is a constant [1].

The RALF model was derived from the Lattice Fluid model of Sanchez and Lacombe [13,14,15] and the following expression for the residual Gibbs energy for fluid was obtained in [1]

(2) $$\begin{array}{ll} \frac{G^{R}\left(T,P,N\right)}{RT}= & rN\left[-\frac{\tilde{\rho }}{\tilde{T}}+\frac{\left(1-\tilde{\rho }\right)\ln\left(1-\tilde{\rho }\right)}{\tilde{\rho }}+1\right]\\ & +N\tilde{\rho }\sum_{j}x_{j}\ln\left(\frac{\phi_{j}}{x_{j}}\right)+N\left(z-1-\ln z\right) \end{array}$$

with → $\tilde{\rho }=\frac{\rho }{\rho^{*}}$ → → $\tilde{T}=\frac{T}{T^{*}}$ → → $z=\frac{PV}{NRT}=r\frac{\tilde{P}}{\tilde{\rho }\tilde{T}}$ → → $\tilde{P}=\frac{P}{P^{*}}$. where $\rho^{*}$ is the characteristic density; $T^{*}$ is the characteristic temperature, related to an energy; $P^{*}$ is the characteristic pressure, related to an energy density; $\phi_{j}$ are the volume fractions of each specie; $r_{j}$ are the number of lattice sites occupied by each specie while r is the same for the mixture.

The following relationship links the characteristic parameters

(3) $$P^{*}v^{*}=RT^{*}$$

Equation (2) has a +1 in the first parenthesis [16] included so that the first term cancels in the limit of zero pressure. The combinatorial term is pre-multiplied by $\tilde{\rho }$ to ensure that at zero pressure the ideal gas state is recovered [1]. While these are minor changes from the NELF formulation, the basis for the RALF model is to define Equation (2) for the adsorbed phase. First the volume occupied by adsorbed molecules at close packing, $v_{i}^{*}$, is corrected to take into account confinement constraints.

(4) $$v_{iA}^{*}=(1+\xi_{iA})v_{i}^{*}\;\to \;\to \;\mathrm{and}\;\to \;\to \;\rho_{iA}^{*}=\frac{\rho_{i}^{*}}{1+\xi_{iA}}$$

The second important modification is that the combinatorial contribution needs to reflect the fact that due to the rigid nature of the solid it does not contribute to the combinatorial term, but has the effect of reducing the volume available to the molecules in the lattice. Therefore, in the original RALF framework, the residual Gibbs energy of the adsorbed phase was specified as

(5) $$\begin{array}{ll} \frac{G_{A}^{R}\left(T,P,N\right)}{RT}= & rN\left[-\frac{\tilde{\rho }}{\tilde{T}}+\frac{\left(1-\tilde{\rho }\right)\ln\left(1-\tilde{\rho }\right)}{\tilde{\rho }}+1\right]\\ & +N\tilde{\rho }\sum_{i}x_{i}\ln\frac{\phi_{i}}{\left(1-\phi_{S}\right)x_{i}}+N\left(z-1-\ln z\right) \end{array}$$

Here, $\left(1-\phi_{S}\right)$ is highlighted to identify the additional deviation from the case of a polymer.

In the model sums over the index i include only the adsorbates, while sums over j include also the solid, which is the last component. In the LF model component, 0 is used for the vacancies.

What is not immediately apparent is the fact that for multicomponent mixtures Equation (5) does not reduce to the Ideal Adsorbed Solution Theory (IAST) [17], which is one of the requirements for thermodynamic consistency [18]. The issue comes from the combinatorial term, which does not cancel as in the limit of zero pressure the reduced density is $\tilde{\rho }={\tilde{\rho }}_{S}=\frac{\rho_{S}}{\rho_{S}^{*}}$ and not zero.

To ensure thermodynamic consistency the residual Gibbs energy of the adsorbed phase has to be corrected so that the combinatorial term is zero at zero pressure and a suitable expression is:

(6) $$\begin{array}{ll} \frac{G_{A}^{R}\left(T,P,N\right)}{RT}= & rN\left[-\frac{\tilde{\rho }}{\tilde{T}}+\frac{\left(1-\tilde{\rho }\right)\ln\left(1-\tilde{\rho }\right)}{\tilde{\rho }}+1\right]\\ & +N\left[\tilde{\rho }-{\tilde{\rho }}_{S}\left(1+\ln\frac{\tilde{\rho }}{{\tilde{\rho }}_{S}}\right)\right]\sum_{i}x_{i}\ln\frac{\phi_{i}}{\left(1-\phi_{S}\right)x_{i}}+\\ & +N\left(z-1-\ln z\right) \end{array}$$

The term multiplying the combinatorial part ensures that both the residual energy and the chemical potential of the adsorbates have the correct limit at zero pressure since

(7) $$\underset{P\to 0}{\lim}\tilde{\rho }-{\tilde{\rho }}_{S}\left(1+\ln\frac{\tilde{\rho }}{{\tilde{\rho }}_{S}}\right)=0$$

This modification does not affect the single adsorbate case as the combinatorial term for a pure adsorbate is zero given that for a single adsorbate $\phi_{1}=\left(1-\phi_{S}\right)$. For mixtures at high adsorbed concentrations the correction is small, thus all numerical results presented in the original RALF model [1] are not affected by this correction.

The mathematical structure of the correction term leads to an equation of state term given by

(8) $$\begin{array}{ll} z_{A}^{EoS}-1= & r\left[-\frac{\tilde{\rho }}{\tilde{T}}-\frac{\ln\left(1-\tilde{\rho }\right)}{\tilde{\rho }}-1\right]\\ & +\left(\tilde{\rho }-{\tilde{\rho }}_{S}\right)\sum_{i}x_{i}\ln\frac{\phi_{i}}{x_{i}\left(1-\phi_{S}\right)}\; \end{array}$$

for which the combinatorial term of the adsorbed phase cancels in the limit of zero pressure.

To apply Equation (6), mixing rules for the model parameters need to be specified. These are [1]

(9) $$r_{i}v^{*}=r_{i}^{0}v_{iA}^{*}$$

which conserves the close-packed molecular volume of each component;

(10) $$\sum_{j}r_{j}^{0}N_{j}=rN$$

which preserves the number of pair interactions in the close-packed state; and

(11) $$P^{*}=\sum_{j}\sum_{k}\phi_{j}\phi_{k}P_{jk}^{*}$$

with → $P_{jk}^{*}=P_{kj}^{*}=\left(1-\kappa_{kj}\right)\sqrt{P_{k}^{*}P_{j}^{*}}$→ and $\kappa_{kk}=0$. which is the classical quadratic mixing rule [19].

The reduced residual chemical potentials can be obtained by derivation

(12) $$\frac{\mu_{k}^{R}}{RT}=\frac{1}{RT}{\left(\frac{\partial G_{A}^{R}}{\partial N_{k}}\right)}_{T,P,N_{j\neq k}}=\ln\varphi_{k}\;\to \;\to \;\to \;\frac{\mu_{S}^{Rm}}{RT}=\frac{1}{RT}{\left(\frac{\partial G_{A}^{R}}{\partial m_{S}}\right)}_{T,P,N_{j\neq S}}$$

where $\varphi_{k}$ is the fugacity coefficient of adsorbate k. Note that an asymmetric convention is used with moles for the adsorbates and mass for the adsorbent.

The expressions for the reduced residual chemical potential of the adsorbates in the case of a frozen solid are:

(13) $$\begin{array}{ll} \frac{\mu_{k}^{R}}{RT}= & -\frac{\tilde{\rho }}{\tilde{T}}r_{k}\left(2\frac{\sum_{j}\phi_{j}P_{kj}^{*}}{P^{*}}-1\right)+\left[\frac{\left(1-\tilde{\rho }\right)\ln\left(1-\tilde{\rho }\right)}{\tilde{\rho }}+1\right]r_{k}^{0}\\ & +\frac{r_{k}}{r}\left(z_{A}^{EoS}-1\right)\\ & +\left[\tilde{\rho }-{\tilde{\rho }}_{S}\left(1+\ln\frac{\tilde{\rho }}{{\tilde{\rho }}_{S}}\right)\right](\ln\frac{r_{k}}{r\left(1-\phi_{S}\right)}+1\\ & -\frac{r_{k}}{r\left(1-\phi_{S}\right)})-\ln z \end{array}$$

Equation (13) is all that is needed for phase equilibrium calculations but to establish the connection between RALF and RAST what is important is the expression for the reduced residual chemical potential of the solid, which for a frozen solid is given by:

(14) $$\frac{\mu_{S}^{Rm}}{RT}=\frac{\tilde{\rho }\phi_{S}}{\rho_{S}v_{S}^{*}}\left[\frac{\left(1-\tilde{\rho }\right)\ln\left(1-\tilde{\rho }\right)}{\tilde{\rho }}+1\right]-\frac{\tilde{\rho }}{\tilde{T}}\left[2\frac{\sum_{j}\phi_{j}P_{Sj}^{*}}{P^{*}}-1\right]\frac{\tilde{\rho }\phi_{S}}{\rho_{S}v^{*}}\;+n\left(\phi_{S}-1\right)\left(z_{A}^{EoS}-1\right)-n+\frac{P}{RT\rho_{S}}$$

where $n=\frac{N}{m_{S}}$ is the total absolute adsorbed amount in moles/kg of adsorbent and $\frac{P}{RT\rho_{S}}=nz$.

From Equation (14) it is possible to define also the reference state for the solid as that of the solid phase without adsorbates at the same pressure of the system [20] as in this limit $\phi_{S}=1$; $\tilde{\rho }={\tilde{\rho }}_{S}$; $v^{*}=v_{S}^{*}$ and N=0, therefore

(15) $$\frac{\mu_{S0}^{Rm}}{RT}=\frac{1}{v_{S}^{*}\rho_{S}^{*}}\left[\frac{\left(1-{\tilde{\rho }}_{S}\right)\ln\left(1-{\tilde{\rho }}_{S}\right)}{{\tilde{\rho }}_{S}}+1-\frac{{\tilde{\rho }}_{S}}{{\tilde{T}}_{S}}\right]+\frac{P}{RT\rho_{S}}$$

These last two expressions lead to the reduced grand potential [20]

(16) $$\begin{array}{ll} \psi =\frac{\mu_{S0}^{Rm}}{RT}-\frac{\mu_{S}^{Rm}}{RT} & \\ & =\frac{1}{v_{S}^{*}\rho_{S}^{*}}\left[\frac{\left(1-{\tilde{\rho }}_{S}\right)\ln\left(1-{\tilde{\rho }}_{S}\right)}{{\tilde{\rho }}_{S}}+1-\frac{{\tilde{\rho }}_{S}}{{\tilde{T}}_{S}}\right]\\ & -\frac{\tilde{\rho }\phi_{S}}{\rho_{S}v_{S}^{*}}\left[\frac{\left(1-\tilde{\rho }\right)\ln\left(1-\tilde{\rho }\right)}{\tilde{\rho }}+1\right]\\ & +\frac{\tilde{\rho }}{\tilde{T}}\left[2\frac{\sum_{j}\phi_{j}P_{Sj}^{*}}{P^{*}}-1\right]\frac{\tilde{\rho }\phi_{S}}{\rho_{S}v^{*}}-n\left(\phi_{S}-1\right)\left(z_{A}^{EoS}-1\right)\\ & +n \end{array}$$

which is a fundamental quantity [11] in the thermodynamics of the adsorbed phase as it allows to define the reference state relative to which activity coefficients can be obtained. One should note that Equation (15) is only a function of composition and the total adsorbed amount and does not depend explicitly on the system pressure.

For completeness and to be able to reproduce all the terms needed in the calculations Table 1 includes all the steps and expressions required to calculate the reduced residual potentials of the adsorbates and the reduced grand potential in the RALF model for the frozen solid. In what follows the numerical calculations will be based on the parameters for silicalite obtained following the procedure in [1] from experimental data including Henry law constants, adsorption energies and pure component isotherms for several adsorbates including normal alkanes [21,22,23].

## 3. Thermodynamic Consistency of an Adsorbed Phase

Talu and Myers [18] cover in some detail the requirements for consistency of adsorption experimental data. The requirements for experimental data apply equally to the results from a model and are the following [18]:

1. Single-gas adsorption isotherms should reduce to Henry’s law at the limit of zero pressure.
2. Multicomponent isotherms should display continuity with single-gas isotherms.
3. At fixed temperature and pressure, thermodynamically consistent x-y diagrams should intersect the predictions from the IAST at least once.
4. In the limit of zero pressure, the IAST should be obtained.
5. Activity coefficients in the adsorbed phase are a function of composition and the reduced grand potential.

The last condition requires a method to determine the activity coefficients of the adsorbed phase from the RALF model and this will be covered in a separate section.

Condition 1 is fulfilled as the RALF model reduces to a Henry law constant at low pressure [1] or

(17) $$n_{1}=\frac{N_{1}}{m_{S}}=K_{P}\left(T\right)P$$

At infinite dilution and in the limit of low pressure, for a single adsorbate, it is possible to obtain [1]

(18) $$\ln K_{P1}=\ln\frac{1\;kg}{\rho_{S}RT}+\frac{{\tilde{\rho }}_{S}}{RT}r_{1}^{0}v_{1}^{*}2P_{1S}^{*}-\left[\frac{\left(1-{\tilde{\rho }}_{S}\right)\ln\left(1-{\tilde{\rho }}_{S}\right)}{{\tilde{\rho }}_{S}}+1\right]r_{1}^{0}\;+r_{1}^{0}\frac{v_{1}^{*}}{v_{S}^{*}}\left[\frac{\ln\left(1-{\tilde{\rho }}_{S}\right)}{{\tilde{\rho }}_{S}}+1\right]$$

Given that the RALF model is formulated in terms of thermodynamically consistent mixing rules, there is continuity when one component in the mixture becomes very dilute, i.e., a ternary mixture reduces correctly to the binary mixtures and a binary mixture reduces correctly to the single component isotherms, thus condition 2 is automatically fulfilled.

**Figure 1:**
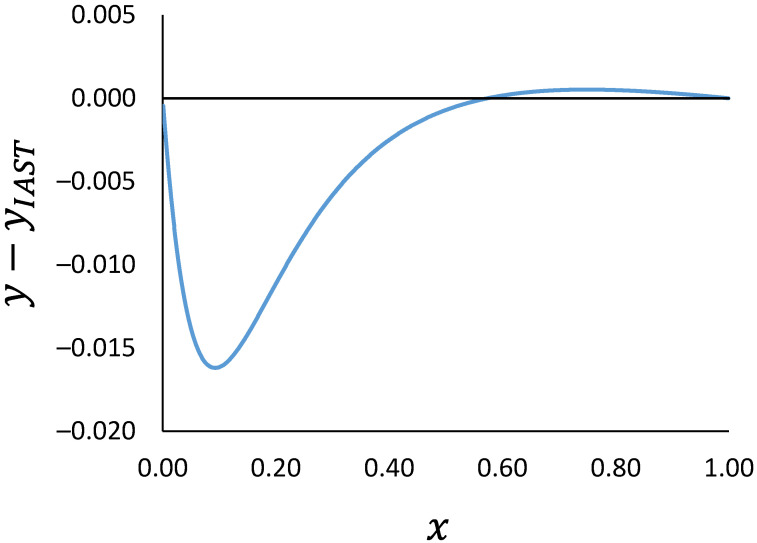
Difference in gas phase mole fraction of ethane calculated from RALF and IAST as a function of adsorbed phase mole fraction. Ethane/n-butane binary mixture at 300 K and 350 kPa.

Condition 3 stems from the integral consistency test and the Gibbs–Duhem equation which requires that at least at one point the activity coefficients of the two components are the same [18]. As a result, the binary selectivity is equal to that of the IAST at least at one point. Figure 1 shows the difference between the gas phase mole fraction calculated from RALF and IAST for the binary ethane/n-butane in silicalite using the RALF parameters reported in [1] at 300 K and 350 kPa. The two models cross at x=0.574. The IAST prediction was obtained using a nested-loop calculation [24] where the reduced grand potential of the ideal mixture, $\psi_{IAST}$, requires the solution of

(19) $$1-\sum_{i=1}^{Nc}\frac{f_{i}^{0}\left(\psi_{IAST}\right)x_{i}}{\varphi_{i}^{F}P}=0$$

where $\varphi_{i}^{F}$ is the fugacity coefficient of each component in the fluid phase and $f_{i}^{0}$ is the reference fugacity calculated from the pure component isotherm. In the RALF model, this last term requires the intermediate calculation of the adsorbed amount for each pure adsorbate from the knowledge of $\psi_{IAST}$. The multicomponent calculations for the RALF model require the solution of the equilibrium relationships given in [1]. For convenience, the fluid phase was described using the Sanchez–Lacombe equation of state modified to be consistent at zero pressure [1].

Condition 4 is fulfilled and this can be demonstrated considering the limit as pressure goes to zero of Equation (13).

(20) $$\frac{\mu_{k}^{R0}}{RT}=-\frac{{\tilde{\rho }}_{S}}{RT}r_{k}^{0}v_{k}^{*}2P_{kS}^{*}+\left[\frac{\left(1-{\tilde{\rho }}_{S}\right)\ln\left(1-{\tilde{\rho }}_{S}\right)}{{\tilde{\rho }}_{S}}+1\right]r_{k}^{0}\;-r_{k}^{0}\frac{v_{k}^{*}}{v_{S}^{*}}\left[\frac{\ln\left(1-{\tilde{\rho }}_{S}\right)}{{\tilde{\rho }}_{S}}+1\right]-\ln z=\ln\left(\frac{1\;kg\sum_{i}n_{i}}{K_{Pk}P}\right)$$

When this is combined with the equilibrium relationship one obtains the correct limiting behaviour of an ideal mixture in the Henry law region

(21) $$n_{k}=K_{Pk}Py_{k}$$

As a final internal thermodynamic consistency check, one can also verify that the reduced grand potential obtained from Equation (16) is consistent with the Gibbs adsorption isotherm [11,25]:

(22) $$\psi =\overset{P}{\underset{0}{\int }}\sum_{i}n_{i}^{A}dlnf_{i}$$

It is relatively straightforward to test this relationship for a single component isotherm and this is a useful check especially in verifying the correct implementation of the RALF model.

## 4. RALF and RAST

In this section, a procedure to obtain the activity coefficients of the adsorbed components from the RALF model will be derived. As the RALF model is a combination of an energy term of a *regular solution* with the combinatorial term of an *athermal solution* [1], one would expect that a simple excess Gibbs energy expression should suffice. The picture in an adsorbed phase is complicated by the fact that the excess Gibbs energy will be a function also of the reduced grand potential. Myers has shown that to a very good approximation $g^{Ex}$ models from liquid phase correlations [19] can be used for the concentration dependence of adsorbed phase provided that an additional term is included [26,27,28]

(23) $$\frac{g^{ExA}}{RT}=\left(1-e^{-C\psi }\right)\frac{g^{Ex}}{RT}=\sum_{i}x_{i}\ln\gamma_{i}^{A}$$

One can see that the additional term ensures that at low pressure the IAST is recovered in compliance with condition 4.

To calculate the activity coefficients of the RALF model one has to consider the definition of the fugacity of the adsorbed phase from RAST, which requires the reference state of the pure components at the same reduced grand potential of the mixture. Therefore, the two adsorbed phase fugacities can be equated

(24) $$\gamma_{k}^{A}\varphi_{k}^{A0}P_{k}^{0}=\varphi_{k}^{A}P$$

This expression can be rearranged to a more convenient form

(25) $$\ln\gamma_{k}^{A}=\frac{\mu_{k}^{R}}{RT}+\ln z-\ln\frac{nRT\rho_{S}}{f_{k}^{A0}}$$

With the pure component reference fugacity $f_{k}^{A0}=\varphi_{k}^{A0}P_{k}^{0}$. Equation (25) depends only on the adsorbed phase concentrations and the total adsorbed amount as is the case for the reduced grand potential.

It is now possible to calculate the excess Gibbs energy by assigning the adsorbed phase mole fractions and the reduced grand potential. The procedure is as follows

6. Determine the total adsorbed amount from Equation (16).
7. Repeat this for the pure component case in order to determine the pure component adsorbed amounts at the same reduced grand potential of the mixture.
8. From the pure component isotherm, calculate the reference pressure and fugacity corresponding to the adsorbed amounts obtained in step 2.
9. Calculate the activity coefficients of all components from Equation (25).

Steps 1 and 2 are fairly easy to implement as each corresponds to the solution of a single nonlinear equation with a function that increases monotonically with the number of moles adsorbed. The physically valid range of the adsorbed amounts will lie between 0 and the finite saturation capacity values for the mixture and for each pure component, respectively. The saturation capacities are obtained by inspection and from the definition of the close-packed state:

(26) $$n^{Sat}\sum_{i}\frac{x_{i}Mw_{i}}{\rho_{iA}^{*}}=\frac{1}{\rho_{S}}\left(1-\frac{\rho_{S}}{\rho_{S}^{*}}\right)$$

For a single adsorbent [1]

(27) $$n_{1}^{Sat}=\frac{1}{Mw_{1}}\frac{\rho_{1A}^{*}}{\rho_{S}}\left(1-\frac{\rho_{S}}{\rho_{S}^{*}}\right)$$

Step 3 is slightly more complicated as the adsorption isotherm is an implicit function of pressure, but the adsorbed amount increases monotonically with pressure and this makes the numerical solution robust.

Spanning the compositions at a fixed reduced grand potential is the most convenient way to see the composition dependence of the excess Gibbs energy of the adsorbed phase. An alternative is to assign increasing values of the total adsorbed amount and determine at a fixed mole fraction the dependence with respect to the reduced grand potential. In this case, Step 1 is replaced by direct computation of the reduced grand potential of the mixture having specified the total amount adsorbed and composition of the adsorbates.

While a detailed investigation of all possible cases that can be generated using the RALF model including the use of the mixture parameters is beyond the scope of this study, here it is possible to investigate the behaviour of the binary mixtures of alkanes in silicalite, which should be represented well by the Non-Ideal Adsorbed Solution Theory, NIAST, of Myers [28]. NIAST is a predictive model based on the simplest expression for activity coefficients, which at a fixed temperature can be written as

(28) $$\frac{g^{ExA}}{RT}=-4\delta_{12}x_{1}x_{2}\left(1-e^{-C_{12}\psi }\right)$$

With

(29) $$\delta_{12}=\left|\frac{\psi_{1}^{0.5}}{n_{1}^{Sat}}-\frac{\psi_{2}^{0.5}}{n_{2}^{Sat}}\right|\;;\to \;C_{12}=\frac{e^{-\delta_{12}}}{n_{12}^{Sat}}\;;\to \;\frac{1}{n_{12}^{Sat}}=\frac{1}{2}\left(\frac{1}{n_{1}^{Sat}}+\frac{1}{n_{2}^{Sat}}\right)$$

$\psi_{k}^{0.5}$ is the reduced grand potential of pure component k at an adsorbed concentration of $0.5n_{k}^{Sat}$. Therefore, the NIAST parameters can be calculated from the pure component isotherms of the RALF model providing an alternative means of predicting the binary mixtures.

**Figure 2:**
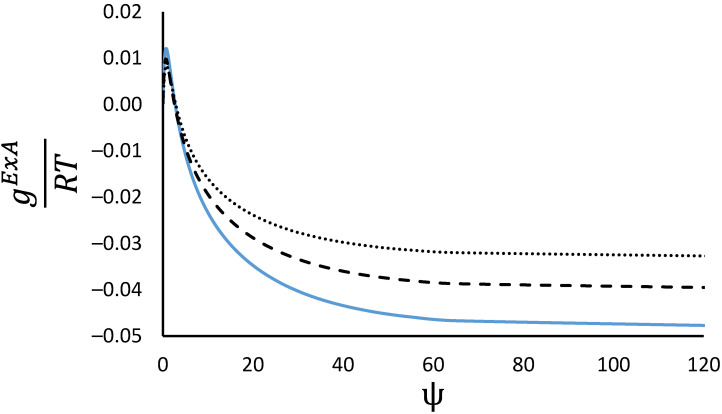
Reduced excess Gibbs energy as a function of the reduced grand potential at mole fractions of methane of 0.25 (dotted line), 0.5 (continuous line) and 0.75 (dashed line). Methane/n-butane binary mixture.

As the deviations from ideality are mild in the alkanes silicalite systems considered, calculations were carried out on the methane/n-butane mixture which shows the largest deviations. Figure 2 shows the excess Gibbs energy predicted at three mole fractions (0.25, 0.5 and 0.75) as a function of the reduced grand potential of the mixture at 300 K.

**Figure 3:**
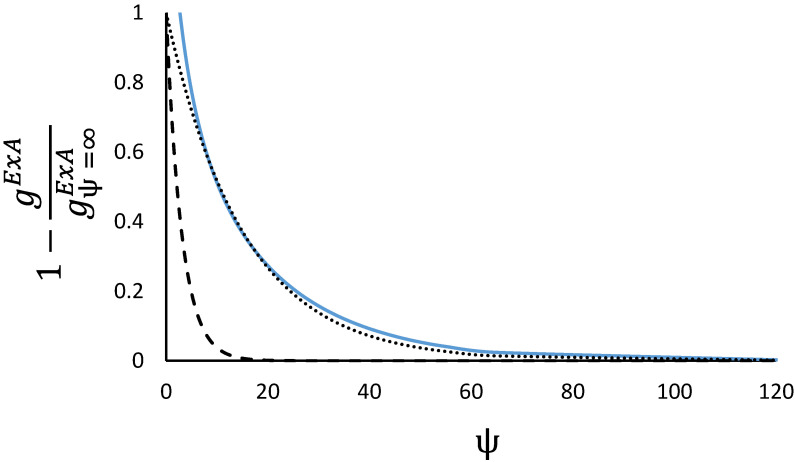
Normalised reduced excess Gibbs energy as a function of the reduced grand potential at a mole fraction of methane of 0.5. RALF predictions for the binary methane/n-butane (continuous line) are compared to simple exponential decay curves with C=0.066 (dotted line) and the predicted NIAST $C_{12}=0.328$ (dashed line).

As can be seen from Figure 2 the RALF model shows a more complex behaviour than the NIAST approach, as the excess Gibbs energy includes some asymmetry that is more pronounced as the adsorbed amount increases (at the same reduced grand potential the curves at 0.25 and 0.75 should overlap in the case of symmetry), and there is also a small region at low reduced grand potential (low pressure and adsorbed amount region) where positive deviations are predicted. Overall, though negative deviations are predicted where typical experimental measurements are carried out. To see more clearly the exponential dependence with respect to the reduced grand potential, Figure 3 shows the normalised excess Gibbs energy for a mole fraction of 0.5.

Structurally the RALF model contains the correct dependence on the reduced grand potential but the predictions are of smaller deviations from ideality at lower pressures compared to the NIAST formulation.

**Figure 4:**
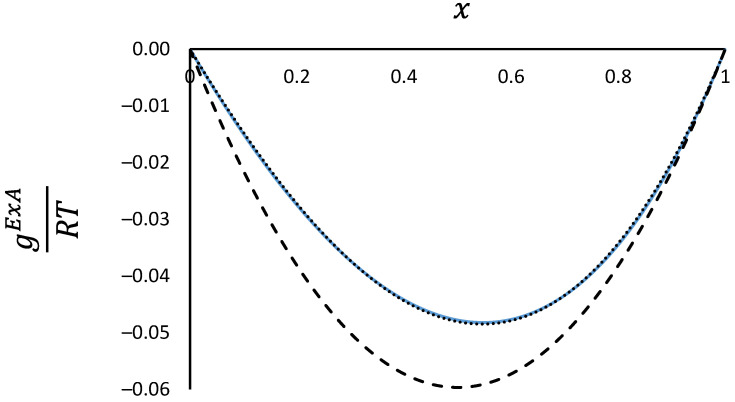
Normalised reduced excess Gibbs energy as a function of the mole fraction of methane at a fixed reduced grand potential ψ=150 mol/kg. RALF prediction (continuous line) and two-parameter Margules equation (dotted line) are compared with NIAST predictions (dashed line) based on RALF pure component isotherms.

Figure 4 shows the comparison at high adsorbed amounts for ψ=150 mol/kg. It is very interesting to note that both NIAST and RALF models predict the same infinite dilution activity coefficient for n-butane in methane (region close to x=1), while RALF predicts a slightly smaller infinite dilution activity coefficient at the other limit, with a resulting asymmetric excess Gibbs energy that can be matched well with a two-parameter Margules binary activity coefficient expression [29] (dotted line). Including the effect of the reduced grand potential the approximate expression is given by

(30) $$\frac{g^{ExA}}{RT}=-\left(A_{21}x_{1}+A_{12}x_{2}\right)x_{1}x_{2}\left(1-e^{-C\psi }\right)$$

Matching the excess Gibbs energy at high reduced grand potential $A_{21}=0.156$ and $A_{12}=0.229$.

**Figure 5:**
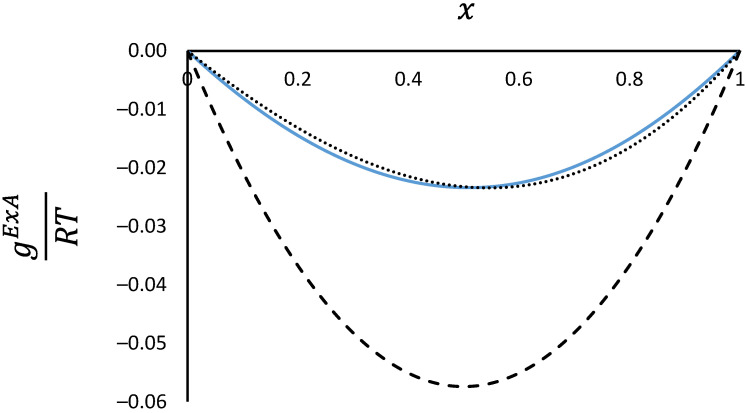
Normalised reduced excess Gibbs energy as a function of the mole fraction of methane at a fixed reduced grand potential ψ=10 mol/kg. RALF prediction (continuous line) and two-parameter Margules equation (dotted line) are compared with NIAST predictions (dashed line) based on RALF pure component isotherms.

At lower reduced grand potential values, the excess Gibbs energy predicted from RALF becomes nearly symmetric as shown in Figure 5 for ψ=10 mol/kg. The dotted line close to the RALF data points is calculated using Equation (29) and the deviation is small.

Structurally RALF is a rather complex model that produces the correct mixture behaviour for the systems considered with similar quantitative results when compared to the NIAST of Myers [28]. The key advantage of RALF is that it is formulated as a multicomponent model and is not limited to binary systems [1].

## 5. Conclusions

A correction to the reduced density function that multiplies the combinatorial term in RALF was shown to lead to a formulation that passes all thermodynamic consistency checks. As the Sanchez–Lacombe equation of state was derived starting from the close-packed limit it was important to carry out these checks and identify the incorrect zero pressure limit of the original formulation.

A procedure to calculate the predicted adsorbed mixture activity coefficients has been presented that allows for the production of excess Gibbs energy plots at a constant reduced grand potential. This calculation does not require the knowledge of the pressure of the system as the problem can be solved by specifying the mole fractions of the adsorbed phase and the total adsorbed amount.

Results from the NIAST model of Myers indicate that the RALF model for frozen solids contains the correct structure in terms of the dependence of non-ideality with respect to composition and the reduced grand potential. In addition to providing the means to analyse the RALF model, the procedure presented can also be used to link the new framework to the traditional non-ideal adsorbed phase approach if an excess Gibbs energy generated from RALF can be matched to a suitable RAST model.

## Figures and Tables

**Table 1:** RALF frozen solid multicomponent steps and expressions.

Sequence	
1	$\mathrm{Specify}\;\mathrm{parameters}:\;T_{i}^{*}$ $;\;P_{i}^{*}$ $;\;\rho_{i}^{*}$ $,\;Mw_{i}$ $;\;T_{S}^{*}$ $;\;P_{S}^{*}$ $;\;\rho_{S}^{*}$ $\;\mathrm{and}\;\kappa_{ij}$
2	$\mathrm{Specify}\;\mathrm{variables}:\;T;\;P;\;n_{i}$ $;\;m_{S}$
3	$v_{S}^{*}=\frac{RT_{S}^{*}}{P_{S}^{*}}$
4	$P_{iA}^{*}=\frac{P_{i}^{*}}{1+\xi_{iA}}$ $\;;\;v_{iA}^{*}=\frac{RT_{i}^{*}}{P_{iA}^{*}}$ $;\;\rho_{iA}^{*}=\frac{\rho_{i}^{*}}{1+\xi_{iA}}$ $;\;r_{iA}^{0}=\frac{Mw_{i}}{v_{iA}^{*}\rho_{iA}^{*}}$
5	$\mathrm{Define}\;m_{i}=n_{i}Mw_{i}$ $\;\mathrm{and}\;m_{T}=\underset{j}{\sum }m_{j}$
6	$\rho^{*}=\frac{m_{T}}{\sum_{i}\frac{m_{i}}{\rho_{iA}^{*}}+\frac{m_{S}}{\rho_{S}^{*}}}$ $\;;\;\phi_{i}=\frac{m_{i}}{\rho_{iA}^{*}}\frac{\rho^{*}}{m_{T}}$ $\;;\;\phi_{S}=\frac{m_{S}}{\rho_{S}^{*}}\frac{\rho^{*}}{m_{T}}$ $\;;\;\rho =\frac{m_{T}}{m_{S}}\rho_{S}$
7	$P^{*}=\underset{j}{\sum }\underset{k}{\sum }\phi_{j}\phi_{k}P_{jk}^{*}$$\;\mathrm{with}\;P_{jk}^{*}=P_{kj}^{*}=\left(1-\kappa_{kj}\right)\sqrt{P_{k}^{*}P_{j}^{*}}$ → $\;\mathrm{and}\;\kappa_{kk}=0$.
8	$\frac{1}{v^{*}}=\underset{j}{\sum }\frac{\phi_{j}}{v_{j}^{*}}$ $\;;\;T^{*}=\frac{P^{*}v^{*}}{R}$ $;\;\tilde{T}=\frac{T}{T^{*}}$ $;\;\tilde{P}=\frac{P}{P^{*}}$ $\;\mathrm{and}\;\tilde{\rho }=\frac{\rho }{\rho^{*}}$
9	$r_{i}=r_{iA}^{0}\frac{v_{iA}^{*}}{v^{*}}$ $\;;\;\frac{1}{r}=\underset{i}{\sum }\frac{\phi_{i}}{r_{i}}$ $\;;\;rN=\frac{m_{S}}{\rho_{S}}\frac{\tilde{\rho }}{v^{*}}$ $\;\mathrm{and}\;z=r\frac{\tilde{P}}{\tilde{\rho }\tilde{T}}$
10	$x_{i}=r\frac{\phi_{i}}{r_{i}}$ $\;\mathrm{and}\;z_{A}^{EoS}-1=r\left[-\frac{\tilde{\rho }}{\tilde{T}}-\frac{\ln\left(1-\tilde{\rho }\right)}{\tilde{\rho }}-1\right]+\left(\tilde{\rho }-{\tilde{\rho }}_{S}\right)\underset{j}{\sum }x_{j}\ln\frac{\phi_{j}}{x_{j}\left(1-\phi_{S}\right)}\;$
$\frac{\mu_{k}^{R}}{RT}=-\frac{\tilde{\rho }}{\tilde{T}}r_{k}\left(2\frac{\sum_{j}\phi_{j}P_{kj}^{*}}{P^{*}}-1\right)+\left[\frac{\left(1-\tilde{\rho }\right)\ln\left(1-\tilde{\rho }\right)}{\tilde{\rho }}+1\right]r_{k}^{0}+\frac{r_{k}}{r}\left(z_{A}^{EoS}-1\right)-\ln z+\left[\tilde{\rho }-{\tilde{\rho }}_{S}\left(1+\ln\frac{\tilde{\rho }}{{\tilde{\rho }}_{S}}\right)\right]\left(\ln\frac{r_{k}}{r\left(1-\phi_{S}\right)}+1-\frac{r_{k}}{r\left(1-\phi_{S}\right)}\right)$ $\Psi =\frac{\mu_{S0}^{Rm}}{RT}-\frac{\mu_{S}^{Rm}}{RT}=\frac{1}{v_{S}^{*}\rho_{S}^{*}}\left[\frac{\left(1-{\tilde{\rho }}_{S}\right)\ln\left(1-{\tilde{\rho }}_{S}\right)}{{\tilde{\rho }}_{S}}+1-\frac{{\tilde{\rho }}_{S}}{{\tilde{T}}_{S}}\right]-\frac{\phi_{S}}{\rho_{S}}\frac{\tilde{\rho }}{v_{S}^{*}}\left[\frac{\left(1-\tilde{\rho }\right)\ln\left(1-\tilde{\rho }\right)}{\tilde{\rho }}+1\right]+\frac{\tilde{\rho }}{\tilde{T}}\left[2\frac{\sum_{j}\phi_{j}P_{Sj}^{*}}{P^{*}}-1\right]\frac{\phi_{S}}{\rho_{S}}\frac{\tilde{\rho }}{v^{*}}-n\left(\phi_{S}-1\right)\left(z^{Eos}-1\right)+n$	

## Data Availability

Not applicable.

## Glossary

f	fugacity (Pa)
$G^{R}$	residual Gibbs energy (J)
$G_{A}^{R}$	residual Gibbs energy of the adsorbed phase (J)
K	dimensionless Henry law constant (–)
$K_{P}$	Henry law constant (mol kg^–1^ Pa^–1^)
$n_{i}$	adsorbed amount of component i (mol kg^–1^)
$n_{i}^{sat}$	saturation capacity of component i (mol kg^–1^)
N	number of moles (mol)
$N_{j}$	number of moles of species j (mol)
$N_{r}$	size of lattice in mole equivalents (mol)
$m_{j}$	mass of species j (kg)
$m_{S}$	mass of solid (kg)
$Mw_{i}$	molecular mass of species i (kg mol^–1^)
P	pressure (Pa)
$P_{0}$	reference pressure (Pa)
$\tilde{P}$	reduced pressure (–)
$P^{*}$	characteristic pressure of the mixture (Pa)
$P_{i}^{*}$	characteristic pressure of component i pure (Pa)
$P_{iA}^{*}$	characteristic pressure of component i in the adsorbed phase (Pa)
$P_{S}^{*}$	characteristic pressure of the solid (Pa)
$P_{ij}^{*}$	pair characteristic pressure (Pa)
r	average number of mers in a molecule (–)
$r_{j}$	number of mers in molecule j in the mixture (–)
$r_{j}^{0}$	number of mers in molecule j pure (–)
R	ideal gas constant (J mol^–1^ K^–1^)
T	temperature (K)
$\tilde{T}$	reduced temperature (–)
$T^{*}$	characteristic temperature of the mixture (K)
$T_{i}^{*}$	characteristic temperature of component i pure (K)
$T_{S}^{*}$	characteristic temperature of the solid (K)
$\tilde{v}$	reduced molar volume (–)
$v^{*}$	average close-packed volume of mers in a mixture (m^3^ mer-mol^–1^)
$v_{j}^{*}$	close-packed volume of mers molecule j pure (m^3^ mer-mol^–1^)
$v_{jA}^{*}$	close-packed volume of mers molecule j in the adsorbed phase (m^3^ mer-mol^–1^)
V	volume of the lattice (m^3^)
$V^{*}$	close-packed volume for the mixture (m^3^)
$V_{S}$	volume of the solid (m^3^)
$w_{j}$	mass fraction of species j (–)
$x_{i}$	mole fraction of species i in the adsorbed phase (–)
$y_{i}$	mole fraction of species i in the fluid phase (–)
z	compressibility factor (–)
$z^{EoS}$	compressibility factor derived from the Helmholtz energy (–)

## Greek Letters

$\beta_{T}$	isothermal solid compressibility (Pa^–1^)
$\Delta H_{0}$	adsorption enthalpy at zero loading (J mol^–1^)
$\Delta U_{0}$	adsorption energy at zero loading (J mol^–1^)
$\varphi_{i}$	fugacity coefficient of species i (–)
$\phi_{j}$	volume fraction in the lattice occupied by species j at close-packing (–)
$\phi_{S}$	volume fraction in the lattice occupied by the solid at close-packing (–)
$\phi_{j}^{L}$	volume fraction in the lattice occupied by species j (–)
$\kappa_{ij}$	pair interaction coefficient (–)
$\mu_{i}^{R}$	residual chemical potential of species i in the adsorbed phase (J mol^–1^)
$\mu_{iF}^{R}$	residual chemical potential of species i in the fluid phase (J mol^–1^)
$\mu_{i\infty }^{R}$	residual chemical potential of species i at infinite dilution in the adsorbed phase (J mol^–1^)
$\mu_{S}^{Rm}$	residual chemical potential of the solid on a mass basis (J kg^–1^)
$\mu_{S0}^{Rm}$	residual chemical potential of the solid without adsorbates (J kg^–1^)
$\tilde{\rho }$	reduced mass density (–)
$\rho^{*}$	average close-packed mass density in a mixture (kg m^–3^)
$\rho_{j}^{*}$	close-packed mass density of molecule j (kg m^–3^)
$\rho_{jA}^{*}$	close-packed mass density of molecule j in the adsorbed phase (kg m^–3^)
$\rho_{S}$	mass density of the solid (kg m^–3^)
$\xi_{jA}$	volume correction due to confinement constraints (–)
Ψ	reduced grand potential (mol kg^–1^)

## References

[1] Brandani S. (2018). The rigid adsorbent lattice fluid model for pure and mixed gas adsorption. AIChE J..

[2] Doghieri F., Sarti G.C. (1996). Nonequilibrium lattice fluids:  A predictive model for the solubility in glassy polymers. Macromolecules.

[3] Sarti G.C., Doghieri F. (1998). Predictions of the solubility of gases in glassy polymers based on the NELF model. Chem. Eng. Sci..

[4] Baschetti M.G., Doghieri A.F., Sarti G.C. (2001). Solubility in Glassy Polymers: Correlations through the Nonequilibrium Lattice Fluid Model. Ind. Eng. Chem. Res..

[5] De Angelis M.G., Sarti G.C., Doghieri F. (2007). Correlations between Penetrant Properties and Infinite Dilution Gas Solubility in Glassy Polymers: NELF Model Derivation. Ind. Eng. Chem. Res..

[6] Minelli M., Sarti G.C. (2019). 110th Anniversary: Gas and Vapor Sorption in Glassy Polymeric Membranes—Critical Review of Different Physical and Mathematical Models. Ind. Eng. Chem. Res..

[7] Verbraeken M.C., Brandani S. (2019). Predictions of Stepped Isotherms in Breathing Adsorbents by the Rigid Adsorbent Lattice Fluid. J. Phys. Chem. C.

[8] Verbraeken M.C., Brandani S. (2019). A priori predictions of type I and type V isotherms by the rigid adsorbent lattice fluid. Adsorption.

[9] Verbraeken M.C., Mennitto R., Georgieva V.M., Bruce E.L., Greenaway A.G., Cox P.A., Min J.G., Hong S.B., Wright P.A., Brandani S. (2020). Understanding CO2 adsorption in a flexible zeolite through a combination of structural, kinetic and modelling techniques. Sep. Purif. Technol..

[10] Brandani S., Mangano E., Santori G. (2022). Water Adsorption on AQSOA-FAM-Z02 Beads. J. Chem. Eng. Data.

[11] Myers A.L., Monson P.A. (2014). Physical adsorption of gases: The case for absolute adsorption as the basis for thermodynamic analysis. Adsorption.

[12] Brandani S., Mangano E., Sarkisov L. (2016). Net, excess and absolute adsorption and adsorption of helium. Adsorption.

[13] Sanchez I.C., Lacombe R.H. (1976). An elementary molecular theory of classical fluids. Pure fluids. J. Phys. Chem..

[14] Lacombe R.H., Sanchez I.C. (1976). Statistical thermodynamics of fluid mixtures. J. Phys. Chem..

[15] Sanchez I.C., Lacombe R.H. (1978). Statistical Thermodynamics of Polymer Solutions. Macromolecules.

[16] Neau E. (2002). A consistent method for phase equilibrium calculation using the Sanchez–Lacombe lattice–fluid equation-of-state. Fluid Phase Equilibria.

[17] Myers A.L., Prausnitz J.M. (1965). Thermodynamics of mixed-gas adsorption. AIChE J..

[18] Talu O., Myers A.L. (1988). Rigorous thermodynamic treatment of gas adsorption. AIChE J..

[19] Prausnitz J.M., Lichtenthaler R.N., de Azevedo E.G. (1999). Molecular Thermodynamics of Fluid-Phase Equilibria.

[20] Brandani S., Mangano E., Luberti M. (2017). Net, excess and absolute adsorption in mixed gas adsorption. Adsorption.

[21] Golden T., Sircar S. (1994). Gas Adsorption on Silicalite. J. Colloid Interface Sci..

[22] Hufton J.R., Danner R.P. (1993). Chromatographic study of alkanes in silicalite: Equilibrium properties. AIChE J..

[23] Abdul-Rehman H.B., Hasanain M.A., Loughlin K.F. (1990). Quaternary, ternary, binary, and pure component sorption on zeolites. Light alkanes on Linde S-115 silicalite at moderate to high pressures. Ind. Eng. Chem. Res..

[24] Mangano E., Friedrich D., Brandani S. (2014). Robust algorithms for the solution of the ideal adsorbed solution theory equations. AIChE J..

[25] Ruthven D.M. (1984). Principles of Adsorption and Adsorption Processes.

[26] Talu O., Li J., Myers A.L. (1995). Activity coefficients of adsorbed mixtures. Adsorption.

[27] Siperstein F.R., Myers A.L. (2001). Mixed-gas adsorption. AIChE J..

[28] Myers A.L. (2005). Prediction of Adsorption of Nonideal Mixtures in Nanoporous Materials. Adsorption.

[29] Smith J.M., Van Ness H.C., Abbott M.M. (1996). Introduction to Chemical Engineering Thermodynamics.

